# Supplementary material for: Surface Water Microplastics in the St. Lawrence River and Estuary in Canada
Source: PLoS One. 2025 Apr 28;20(4):e0315739. doi: 10.1371/journal.pone.0315739 (PMC12036840; doi:10.1371/journal.pone.0315739)
Supplement: S1 Table — (DOCX) [file pone.0315739.s001.docx]

**Table S1.** Akaike Information Criterion on small sample size (AICc) for competing models ranked from most to least parsimonious with area, site, and mesh size as independent variables to explain the sum of microplastic particles combined and subsetted for fiber and fragments.

| **Model** | **df** | **AICc** | **ΔAICc** | **weight** |
| --- | --- | --- | --- | --- |
| **All** |  |  |  |  |
| Area | 3 | 57 |  | 0.36 |
| Null | 2 | 57 | 0.36 | 0.30 |
| Mesh size | 3 | 59 | 1.8 | 0.15 |
| Site | 12 | 60 | 3.5 | 0.06 |
| Site + Area | 12 | 60 | 3.5 | 0.06 |
| Site + Area + Mesh size | 13 | 62 | 4.8 | 0.03 |
| Site + Mesh size | 13 | 62 | 4.8 | 0.03 |
| **Fiber** |  |  |  |  |
| Null | 2 | 80 |  | 0.48 |
| Mesh size | 3 | 81 | 0.69 | 0.34 |
| Area | 3 | 83 | 2.11 | 0.17 |
| Site | 12 | 90 | 9.8 | 0.004 |
| Site + Area | 12 | 90 | 9.8 | 0.004 |
| Site + Mesh size | 13 | 91 | 10.1 | 0.003 |
| Site + Area + Mesh size | 13 | 91 | 10.1 | 0.003 |
| **Fragments** |  |  |  |  |
| Area | 3 | 70 |  | 0.31 |
| Site + Area | 12 | 71 | 0.38 | 0.26 |
| Site | 12 | 71 | 0.38 | 0.26 |
| Site + Area +Mesh size | 13 | 74 | 3.3 | 0.06 |
| Site + Mesh size | 13 | 74 | 3.3 | 0.06 |
| Null | 2 | 75 | 4.6 | 0.03 |
| Mesh size | 3 | 77 | 6.3 | 0.01 |
