# Supplementary material for: Surface Water Microplastics in the St. Lawrence River and Estuary in Canada
Source: PLoS One. 2025 Apr 28;20(4):e0315739. doi: 10.1371/journal.pone.0315739 (PMC12036840; doi:10.1371/journal.pone.0315739)
Supplement: S2 Table — Shannon and Simpson diversity index for site and area using the vegan package with function “adonis” (n = 61). Permutations were set at 999. (DOCX) [file pone.0315739.s002.docx]

**Table S2**. Shannon and Simpson diversity index for site and area using the vegan package with function “adonis” (n = 61). Permutations were set at 999.

| **Index** | **Variable** | **F-ratio** | ***R*^2^** | **p** |
| --- | --- | --- | --- | --- |
| Shannon | Site | 0.73 | 0.14 | 0.75 |
|  | Area | 0.99 | 0.02 | 0.37 |
| Simpson | Site | 0.81 | 0.15 | 0.69 |
|  | Area | 1.4 | 0.02 | 0.26 |
