# Supplementary material for: Surface Water Microplastics in the St. Lawrence River and Estuary in Canada
Source: PLoS One. 2025 Apr 28;20(4):e0315739. doi: 10.1371/journal.pone.0315739 (PMC12036840; doi:10.1371/journal.pone.0315739)
Supplement: Attachment — Submitted filename: Langlois et al. PLOS ONE review_clean.docx [file pone.0315739.s003.docx]

1) Summary of manuscript and overall impression:

This study quantified and characterized microplastics in the surface waters of the St. Lawrence River and Estuary (SLRE) using nets with two different mesh sizes, to fill a gap in our understanding of microplastic abundance in the SLRE’s bulk water. The SLRE has been sampled previously for microplastic, but the novelty of this study is that it investigates the relationship between microplastic abundance and salinity. Authors predicted that microplastic concentrations will scale with salinity and urbanness of land use. Microplastics were detected at every site, concentrations of fragments were higher downstream than upstream, and there is some evidence that assemblage structure of MPs varied between fluvial and estuarine portions of the SLRE system, supporting the prediction that salinity plays a role in MP fate.

The question of the relationship between microplastic concentration and salinity is interesting and novel. The statistics and figures presented are also interesting. Most importantly, the science is sound, which is the criterion for publication in PLOS ONE. Recommendation: major revision. However, I recommend the writing be clearer throughout the manuscript, and also authors take the time to explain more details in the methods, results, and discussion sections. There is no word limit, and there are parts of the manuscript that can be flushed out more. Also at this moment, there are methodological details that are missing and preclude replication of this study. In addition, all data need to be available at the time of manuscript submission, not available upon request. See comments below.

1) Questions and comments:

How were blanks collected? E.g. distilled water poured onto net, then resuspended in the same way as samples? How much distilled water was poured onto net for each blank?

Authors say that several digestion methods were tested; please list these methods somewhere in the manuscript or SI. Also remember to refer to the raw data in your repository.

Fiber concentrations inferred using Manta net sampling is not accurate, because Manta nets underestimate particles smaller than its net mesh. For fibers, its diameter is typically 10-20 um, meaning it can slip through the manta net. Please add a line in your manuscript about how your fiber concentrations in this river system should be interpreted with caution because Manta nets are not designed to measure fibers.

Hung et al. (2020): “Capture of these particles is likely by random chance and is expected to be an underestimate of the total number of these small‐sized particles. For this reason, the quantities of particles with dimension smaller than the mesh size should be treated with caution and considered qualitative estimates.”  <https://doi.org/10.1002/ieam.4325>

Also see Zhu et al. (2021): “Two surface water collection methods were employed: manta trawl [(22,28)](javascript:void(0);) with a 333 μm mesh and 1 L grab. Manta samples (*N* = 65) were collected to quantify and characterize all microdebris, except for microfibers, which can slip through the holes in the mesh due to their shape.” <https://pubs.acs.org/doi/full/10.1021/acsestwater.0c00292>

For results section, please add this basic information: average +/- SD for all 11 sites combined, which site had highest MP concentration (average +/- SD), which site had lowest MP concentration (average +/- SD). I’m not sure why you are using this new measure of concentration, “particles of plastic per million liters” or PPML; instead of inventing a new unit, please consider using existing units to make your study more comparable to others. See Bucci et al. (2020): usually, units of concentration used in ecotoxicology tests are “per mL” or “per L”. More and more often, ecotoxicologists are trying to use environmentally relevant concentrations in their experiments. To make their lives easier, and to harmonize reporting across studies, please consider reporting in a unit that other people use. Unless you provide justification for why you are using a new unit. Please report the average +/- SD concentration for your blanks. Did you blank correct?

For methods section: Equation 1 implies that you only counted half the particles on your filter. State this explicitly somewhere in the Methods section that you subsampled. Why – to save time? Because the filters had a lot of particles on them? Please provide justification. How many particles (%) were selected for FTIR analysis? See de Frond et al. (2022) for minimum number of particles that need to be identified in microplastic samples: <https://www.sciencedirect.com/science/article/abs/pii/S0045653522032659?via%3Dihub>

Discussion: please discuss potential limitations of this study

Line by line:

Equation 1: does ‘debit’ mean ‘volume’?

Figure 1: the white is supposed to indicate water, but the US is also shown in white. Please consider making the US a different colour to indicate land.

Figure 3: colours are a bit harsh to the eye, and also they are not colourblind-friendly. Please consider using a different colour palette, e.g. viridis. I verified this using a chromatic vision simulator app – you can download this app yourself and test the colour palette you end up choosing.

Figure 5 caption: “areas are separated by a vertical dashed line”. What areas? fluvial vs estuary? Please add this information to your figure caption; make sure captions in general are more informative.

Line 35: “we assessed MP fraction in the top 40 cm of surface water” is more accurate, no? Right now in the abstract it sounds like you sampled at a depth of 40 cm.

Line 165: state ranges of % fibers and % fragments across the 11 sites, or some other summary statistic

Lines 208-209: cite a source stating that PE and PP are some of the toughest plastics

Line 210: more accurately, photooxidation is only prevalent in oxygenated areas of ocean exposed to ample sunlight such as the ocean surface

Lines 214-216: cite this fact about PS

Lines 233-234: you say concentrations never exceed 0.001 particles per L – for surface water? And where – in US and Canada? Please make your writing a bit clearer, more informative. But in Rowenczyk et al. (2022), they found a maximum of 0.032 MPs per L in SLRE middle portion – contradiction? Also in Rowenczyk et al., they use particles/L. In order to make your results easily comparable to theirs, doesn’t it make sense to use the same units? I have not seen PPML used before in the literature. Two possibilities: 1) PPML has not been used before, then consider not creating a new unit; we already have a harmonization problem in the plastics field. 2) PPML has been used before: even if this is the case, it deviates from the norm and also makes harmonization difficult.
