# Supplementary material for: Surface Water Microplastics in the St. Lawrence River and Estuary in Canada
Source: PLoS One. 2025 Apr 28;20(4):e0315739. doi: 10.1371/journal.pone.0315739 (PMC12036840; doi:10.1371/journal.pone.0315739)
Supplement: Attachment — Submitted filename: Review comments -20240906.docx [file pone.0315739.s004.docx]

The manuscript "Identification of the Driving Factors of Microplastic Load and Morphology in Estuaries for Improving Monitoring and Management Strategies: A Global Meta-analysis" was well organized and provided a comprehensive understanding of the morphological characteristics and polymers of microplastics in the St. Lawrence River and Estuary in Canada. However, there are still some concerns as follows, which did not be clarified in the manuscript. The English language of the manuscript needs to be improved by a native English speaker, particular in there are too many long sentences without commas in the manuscript. Overall, I recommend to publish the manuscript after the minor revisions.

Specific comments

L33-34: Too specific, it should be more macroscopic, the harmful of microplastics are not limited on living organisms.

L37: Confirm if the use of commas is correct.

L41: Please use passive tense while presenting the results. Do not use "our".

L41: It is best to indicate the proportion

L45-46: Please rewrite the sentence.

L50: Please indicate the definition of microplastics when it first appears.

L79-81: Change to “Firstly, secondly”.

L120: How many (proportion) Potential MPs were detected by ATR-FTIR.

L162: First describe the shape, then talk about the polymer, and what about the concentration?

L203: Please bring appropriate references regarding your statement.

L218: Plastics or microplastics; in China or in Chinese river?

L228: In a previous study

L235: Please rewrite the sentence.

Figure 2 lacks a scale bar

Figure 5 can add color to make it look better
