# Supplementary material for: Surface Water Microplastics in the St. Lawrence River and Estuary in Canada
Source: PLoS One. 2025 Apr 28;20(4):e0315739. doi: 10.1371/journal.pone.0315739 (PMC12036840; doi:10.1371/journal.pone.0315739)
Supplement: Attachment — Submitted filename: Langlois et al. Response to Reviews.docx [file pone.0315739.s005.docx]

**Response to Reviews**

**Editor’s comments:**

1. Please ensure that your manuscript meets PLOS ONE's style requirements, including those for file naming. The PLOS ONE style templates can be found at

[https://journals.plos.org/plosone/s/file?id=wjVg/PLOSOne_formatting_sample_main_body.pdf](https://can01.safelinks.protection.outlook.com/?url=https%3A%2F%2Fc05y1x9s.r.us-east-2.awstrack.me%2FL0%2Fhttps%3A%252F%252Fjournals.plos.org%252Fplosone%252Fs%252Ffile%253Fid%3DwjVg%252FPLOSOne_formatting_sample_main_body.pdf%2F1%2F010f019207f2190c-ad588b30-d639-4154-9a1f-f54e3284b7a1-000000%2F1VAjRMPdwP4O8fUUQ8PcoBb5t-c%3D177&data=05%7C02%7Cvalerie.langlois%40inrs.ca%7C875097ef8eea453dfe0a08dcd84c591e%7C80c9267a177f49439958b599ca0e54c2%7C0%7C0%7C638623071606819511%7CUnknown%7CTWFpbGZsb3d8eyJWIjoiMC4wLjAwMDAiLCJQIjoiV2luMzIiLCJBTiI6Ik1haWwiLCJXVCI6Mn0%3D%7C0%7C%7C%7C&sdata=cmJS%2BsqbT8QUyrbVjISAV7dM%2BKJ0E3in3xUD5sNXKys%3D&reserved=0) and

[https://journals.plos.org/plosone/s/file?id=ba62/PLOSOne_formatting_sample_title_authors_affiliations.pdf](https://can01.safelinks.protection.outlook.com/?url=https%3A%2F%2Fc05y1x9s.r.us-east-2.awstrack.me%2FL0%2Fhttps%3A%252F%252Fjournals.plos.org%252Fplosone%252Fs%252Ffile%253Fid%3Dba62%252FPLOSOne_formatting_sample_title_authors_affiliations.pdf%2F1%2F010f019207f2190c-ad588b30-d639-4154-9a1f-f54e3284b7a1-000000%2FzmfPfxnXLZ1RiSk7CacU4g3VR4g%3D177&data=05%7C02%7Cvalerie.langlois%40inrs.ca%7C875097ef8eea453dfe0a08dcd84c591e%7C80c9267a177f49439958b599ca0e54c2%7C0%7C0%7C638623071606825943%7CUnknown%7CTWFpbGZsb3d8eyJWIjoiMC4wLjAwMDAiLCJQIjoiV2luMzIiLCJBTiI6Ik1haWwiLCJXVCI6Mn0%3D%7C0%7C%7C%7C&sdata=MpP5QCM%2Fa4bcUrYaWLyuSCve3Ev9x8OdLjZh%2BEEkMtk%3D&reserved=0)

Done.

2. In your Methods section, please provide additional information regarding the permits you obtained for the work. Please ensure you have included the full name of the authority that approved the field site access and, if no permits were required, a brief statement explaining why.

Done. This sentence was added. *No permits were required to sample water in the SLRE.*

3. Please note that PLOS ONE has specific guidelines on code sharing for submissions in which author-generated code underpins the findings in the manuscript. In these cases, all author-generated code must be made available without restrictions upon publication of the work.

Please review our guidelines at <https://journals.plos.org/plosone/s/materials-and-software-sharing#loc-sharing-code> and ensure that your code is shared in a way that follows best practice and facilitates reproducibility and reuse.

Done. The code is shared on an open access repository on GitHub at <https://github.com/ECCC-lavoie-ecotox/MPs_St-Lawrence> in a way that follows best practice and facilitates reproducibility and reuse. This was added at the end of the Methodology section. As we needed helped for this, we have added this line to our Acknowledgements: We thank Steve Vissault for reviewing R scripts.

4. We note that the grant information you provided in the ‘Funding Information’ and ‘Financial Disclosure’ sections do not match.

 When you resubmit, please ensure that you provide the correct grant numbers for the awards you received for your study in the ‘Funding Information’ section.

Done.

5. Please expand the acronym “CNRS” (as indicated in your financial disclosure) so that it states the name of your funders in full.

This information should be included in your cover letter; we will change the online submission form on your behalf.

Done. CNRS: Centre national de la recherche scientifique. This got added to the cover letter.

6. Thank you for stating the following financial disclosure:

“The authors acknowledge the funding from the Canada Research Chair Program to VSL and from the CNRS to JG.”

Please state what role the funders took in the study. If the funders had no role, please state: "The funders had no role in study design, data collection and analysis, decision to publish, or preparation of the manuscript". If this statement is not correct you must amend it as needed.

Please include this amended Role of Funder statement in your cover letter; we will change the online submission form on your behalf.

Done. We have added your suggested statement to the cover letter as requested.

7. Please note that funding information should not appear in the Acknowledgments section or other areas of your manuscript. We will only publish funding information present in the Funding Statement section of the online submission form. Please remove any funding-related text from the manuscript.

We have ensured to not mention funding in the Acknowledgments.

8. In the online submission form, you indicated that “All data files are available upon request.”.

All PLOS journals now require all data underlying the findings described in their manuscript to be freely available to other researchers, either

1) In a public repository,
2) Within the manuscript itself, or
3) Uploaded as supplementary information.

This policy applies to all data except where public deposition would breach compliance with the protocol approved by your research ethics board. If your data cannot be made publicly available for ethical or legal reasons (e.g., public availability would compromise patient privacy), please explain your reasons on resubmission and your exemption request will be escalated for approval.

We have attached the raw data to this revised submission. Please change the data availability box for us.

9. We note that Figure 1 in your submission contain map images which may be copyrighted. All PLOS content is published under the Creative Commons Attribution License (CC BY 4.0), which means that the manuscript, images, and Supporting Information files will be freely available online, and any third party is permitted to access, download, copy, distribute, and use these materials in any way, even commercially, with proper attribution. For these reasons, we cannot publish previously copyrighted maps or satellite images created using proprietary data, such as Google software (Google Maps, Street View, and Earth). For more information, see our copyright guidelines: <http://journals.plos.org/plosone/s/licenses-and-copyright>.

We require you to either (1) present written permission from the copyright holder to publish these figures specifically under the CC BY 4.0 license, or (2) remove the figures from your submission:

1) You may seek permission from the original copyright holder of Figure 1 to publish the content specifically under the CC BY 4.0 license.

We recommend that you contact the original copyright holder with the Content Permission Form (http://journals.plos.org/plosone/s/file?id=7c09/content-permission-form.pdf) and the following text:

“I request permission for the open-access journal PLOS ONE to publish XXX under the Creative Commons Attribution License (CCAL) CC BY 4.0 (http://creativecommons.org/licenses/by/4.0/). Please be aware that this license allows unrestricted use and distribution, even commercially, by third parties. Please reply and provide explicit written permission to publish XXX under a CC BY license and complete the attached form.”

Please upload the completed Content Permission Form or other proof of granted permissions as an ""Other"" file with your submission.

In the figure caption of the copyrighted figure, please include the following text: “Reprinted from [ref] under a CC BY license, with permission from [name of publisher], original copyright [original copyright year].”

2) If you are unable to obtain permission from the original copyright holder to publish these figures under the CC BY 4.0 license or if the copyright holder’s requirements are incompatible with the CC BY 4.0 license, please either i) remove the figure or ii) supply a replacement figure that complies with the CC BY 4.0 license. Please check copyright information on all replacement figures and update the figure caption with source information. If applicable, please specify in the figure caption text when a figure is similar but not identical to the original image and is therefore for illustrative purposes only.

The following resources for replacing copyrighted map figures may be helpful:

USGS National Map Viewer (public domain): <http://viewer.nationalmap.gov/viewer/>

The Gateway to Astronaut Photography of Earth (public domain): <http://eol.jsc.nasa.gov/sseop/clickmap/>

Maps at the CIA (public domain): <https://www.cia.gov/library/publications/the-world-factbook/index.html> and <https://www.cia.gov/library/publications/cia-maps-publications/index.html>

NASA Earth Observatory (public domain): <http://earthobservatory.nasa.gov/>

Landsat: <http://landsat.visibleearth.nasa.gov/>

USGS EROS (Earth Resources Observatory and Science (EROS) Center) (public domain): [http://eros.usgs.gov/#](http://eros.usgs.gov/)

Natural Earth (public domain): <http://www.naturalearthdata.com/>

Shapefiles were taken from Open Canada, which is compatible with the CC BY 4.0 license. The following statement was included in the caption of Figure 1: *Basemap source: Government of Canada; Natural Resources Canada (*[*https://open.canada.ca/data/en/dataset/8ba2aa2a-7bb9-4448-b4d7-f164409fe056*](https://open.canada.ca/data/en/dataset/8ba2aa2a-7bb9-4448-b4d7-f164409fe056)*) and Natural Earth (public domain):* [*http://www.naturalearthdata.com/*](http://www.naturalearthdata.com/)*. Contains information licensed under the Open Government License – Canada (*[*https://open.canada.ca/en/open-government-licence-canada*](https://open.canada.ca/en/open-government-licence-canada)*) and Natural Earth (*[*https://www.naturalearthdata.com/about/terms-of-use/*](https://www.naturalearthdata.com/about/terms-of-use/)*).*

10. Please include captions for your Supporting Information files at the end of your manuscript, and update any in-text citations to match accordingly. Please see our Supporting Information guidelines for more information: <http://journals.plos.org/plosone/s/supporting-information>.

Data are shared on an open access repository on GitHub at <https://github.com/ECCC-lavoie-ecotox/MPs_St-Lawrence> in a way that follows best practice and facilitates reproducibility and reuse. This was added at the end of the Methodology section.

**Reviewer #1:** Conclusion is supported by the results and methodological approach. Statistical analysis is sound. Manuscript is understandable, but more details need to be added throughout. All data need to be available at the time of manuscript submission, not available upon request. Please provide all underlying datasets used to inform this study.

Thanks for the positive comments. Dataset were added to the submission.

1) Summary of manuscript and overall impression:

This study quantified and characterized microplastics in the surface waters of the St. Lawrence River and Estuary (SLRE) using nets with two different mesh sizes, to fill a gap in our understanding of microplastic abundance in the SLRE’s bulk water. The SLRE has been sampled previously for microplastic, but the novelty of this study is that it investigates the relationship between microplastic abundance and salinity. Authors predicted that microplastic concentrations will scale with salinity and urbanness of land use. Microplastics were detected at every site, concentrations of fragments were higher downstream than upstream, and there is some evidence that assemblage structure of MPs varied between fluvial and estuarine portions of the SLRE system, supporting the prediction that salinity plays a role in MP fate.

The question of the relationship between microplastic concentration and salinity is interesting and novel. The statistics and figures presented are also interesting. Most importantly, the science is sound, which is the criterion for publication in PLOS ONE. Recommendation: major revision. However, I recommend the writing be clearer throughout the manuscript, and also authors take the time to explain more details in the methods, results, and discussion sections. There is no word limit, and there are parts of the manuscript that can be flushed out more. Also at this moment, there are methodological details that are missing and preclude replication of this study. In addition, all data need to be available at the time of manuscript submission, not available upon request. See comments below.

Thanks for the positive comments. Dataset were added to the submission.

1) Questions and comments:

How were blanks collected? E.g. distilled water poured onto net, then resuspended in the same way as samples? How much distilled water was poured onto net for each blank?

A 1-L distilled water was poured into a glass on the boat and put at 4 ^o^C until filtration. This has been specified in the text.

Authors say that several digestion methods were tested; please list these methods somewhere in the manuscript or SI. Also remember to refer to the raw data in your repository.

We have tested the digestion with hydroperoxide, H_2_O_2_ 30 % (v/v) for different times and temperatures: 6 h at 65 ^o^C, 24 h at 50 ^o^C, 24 h at 25 ^o^C, and 4 h at 50 ^o^C with Fe(II).

This was adjusted in the text.

Fiber concentrations inferred using Manta net sampling is not accurate, because Manta nets underestimate particles smaller than its net mesh. For fibers, its diameter is typically 10-20 um, meaning it can slip through the manta net. Please add a line in your manuscript about how your fiber concentrations in this river system should be interpreted with caution because Manta nets are not designed to measure fibers. Hung et al. (2020): “Capture of these particles is likely by random chance and is expected to be an underestimate of the total number of these small‐sized particles. For this reason, the quantities of particles with dimension smaller than the mesh size should be treated with caution and considered qualitative estimates.” <https://doi.org/10.1002/ieam.4325> Also see Zhu et al. (2021): “Two surface water collection methods were employed: manta trawl (22,28) with a 333 μm mesh and 1 L grab. Manta samples (N = 65) were collected to quantify and characterize all microdebris, except for microfibers, which can slip through the holes in the mesh due to their shape.” https://pubs.acs.org/doi/full/10.1021/acsestwater.0c00292

Thanks for pointing this out. We have added your suggested comments to the text and use the Zhu et al. citation. *Caution should also be considered when interpreting fiber MPs caught by the Manta as its underestimate particles smaller than its net mesh [45]. Therefore, the used of smaller mesh size nets like PolyMer (this study) is favored to improve fiber MP estimations.*

For results section, please add this basic information: average +/- SD for all 11 sites combined, which site had highest MP concentration (average +/- SD), which site had lowest MP concentration (average +/- SD). I’m not sure why you are using this new measure of concentration, “particles of plastic per million liters” or PPML; instead of inventing a new unit, please consider using existing units to make your study more comparable to others. See Bucci et al. (2020): usually, units of concentration used in ecotoxicology tests are “per mL” or “per L”. More and more often, ecotoxicologists are trying to use environmentally relevant concentrations in their experiments. To make their lives easier, and to harmonize reporting across studies, please consider reporting in a unit that other people use. Unless you provide justification for why you are using a new unit. Please report the average +/- SD concentration for your blanks. Did you blank correct?

The information on average +/- SD for all 11 sites combined, which site had highest MP concentration (average +/- SD), which site had lowest MP concentration (average +/- SD) was added in the text: *MP concentrations (mean +/- SD) for all 11 sites combined was 72.3 ± 93.0 PPML and was highest at the La Pocatière site (111.0 ± 120.0 PPML) and lowest at the Sorel site (38.7 ± 40.8 PPML).*

This unit (PPML) was simply used because of the small number of MPs in the samples. Otherwise, the numbers would have been too long to report (too many 0.0000). The average +/- SD were added. Yes, we blank corrected.

For methods section: Equation 1 implies that you only counted half the particles on your filter. State this explicitly somewhere in the Methods section that you subsampled. Why – to save time? Because the filters had a lot of particles on them? Please provide justification.

We kept the second halve of the filters to study nanoplastics, which will be part of another project. We have clarified this in the text.

How many particles (%) were selected for FTIR analysis? See de Frond et al. (2022) for minimum number of particles that need to be identified in microplastic samples: https://www.sciencedirect.com/science/article/abs/pii/S0045653522032659?via%3Dihub

All particles were analyzed by FTIR.

Discussion: please discuss potential limitations of this study

We have added the Manta net size limit in catching fibers to the discussion.

Line by line:

Equation 1: does ‘debit’ mean ‘volume’?

Yes. Sorry for this translation issue. It was fixed.

Figure 1: the white is supposed to indicate water, but the US is also shown in white. Please consider making the US a different colour to indicate land.

Water is now blue and land is white.

Figure 3: colours are a bit harsh to the eye, and also they are not colourblind-friendly. Please consider using a different colour palette, e.g. viridis. I verified this using a chromatic vision simulator app – you can download this app yourself and test the colour palette you end up choosing.

We changed the colour palette, so they are colourblind-friendly.

Figure 5 caption: “areas are separated by a vertical dashed line”. What areas? fluvial vs estuary? Please add this information to your figure caption; make sure captions in general are more informative.

Done.

Line 35: “we assessed MP fraction in the top 40 cm of surface water” is more accurate, no? Right now in the abstract it sounds like you sampled at a depth of 40 cm.

Done.

Line 165: state ranges of % fibers and % fragments across the 11 sites, or some other summary statistic

This text was added. *Approximatively, 20 to 85% were fibers, 10 to 70% were fragments, and 0 to 20% were spheres. Overall, more MPs were captured using the finer mesh size.*

Lines 208-209: cite a source stating that PE and PP are some of the toughest plastics

To be more careful, the authors decided to change the wording for this: *In the case of* *polypropylene and polyethylene, their high mechanical resistance and physical properties make them resistant plastics.*

Line 210: more accurately, photooxidation is only prevalent in oxygenated areas of ocean exposed to ample sunlight such as the ocean surface

The sentence was changed accordingly.

Lines 214-216: cite this fact about PS

Done.

Lines 233-234: you say concentrations never exceed 0.001 particles per L – for surface water? And where – in US and Canada? Please make your writing a bit clearer, more informative. But in Rowenczyk et al. (2022), they found a maximum of 0.032 MPs per L in SLRE middle portion –contradiction? Also in Rowenczyk et al., they use particles/L. In order to make your results easily comparable to theirs, doesn’t it make sense to use the same units? I have not seen PPML used before in the literature. Two possibilities: 1) PPML has not been used before, then consider not creating a new unit; we already have a harmonization problem in the plastics field. 2) PPML has been used before: even if this is the case, it deviates from the norm and also makes harmonization.

Thanks for pointing this out, we have revamped the paragraph as: *We expected to find higher MP concentrations in surface waters upstream than downstream considering the high population density living nearby the Great Lakes and SLRE from the United States and Canada, but lower concentrations were found than elsewhere. For example, Luo et al. (2019) measured concentrations around 2,000,000 PPML near major urban centers in China [46]. This difference can be explained by the hydrology and typology of the SLRE. Indeed, studies have shown that shoreline accumulation and deposition in the sediment were not considered as they can be collected and decontaminated [22]. Castañeda et al. (2014) have estimated MP loadings in SLRE sediment up to 3,800,000 PPML [22] at sites which have accumulated continuously for over 50 years compared to transitioning surface water [47]. In another study, fragments and fibers are the most prevalent particles found in the sediment [48] in South Africa, which could correlate with the variation in shape observed from the sampling* campaign.

The second part of the question was addressed above.

**Reviewer #2:** The manuscript "Identification of the Driving Factors of Microplastic Load and Morphology in Estuaries for Improving Monitoring and Management Strategies: A Global Meta-analysis" was well organized and provided a comprehensive understanding of the morphological characteristics and polymers of microplastics in the St. Lawrence River and Estuary in Canada. However, there are still some concerns as follows, which did not be clarified in the manuscript. The English language of the manuscript needs to be improved by a native English speaker, particular in there are too many long sentences without commas in the manuscript. Overall, I recommend publishing the manuscript after the minor revisions.

Thanks for the positive comments. The manuscript has now been revised by a native English speaker, Dr. Scott Hepditch. You can see his track changes in the revised text. We have also thanked him in the Acknowledgements.
**Specific comments**
L33-34: Too specific, it should be more macroscopic, the harmful of microplastics are not limited on living organisms.

This was revised to “most ecosystems”.

L37: Confirm if the use of commas is correct.

Good catch. It was a typo.

L41: Please use passive tense while presenting the results. Do not use "our".

This was revised to “Data”. All other “our” were modified to the passive tense.

L41: It is best to indicate the proportion

Done.

L45-46: Please rewrite the sentence.

Done. It was revised to: *Notably the findings also suggest MPs are more likely aggregating when salinity increases.*

L50: Please indicate the definition of microplastics when it first appears.

The paragraph was modified accordingly. *Microplastics (MPs; size ranging from 5 mm to 1 μm) are recognized as the breakdown products of macroplastics, such as tire abrasion particles or textile washing fibers [1]. Consequently, the substantial proportion of marine macroplastics is anticipated to degrade over time, leading to a putative increase in MPs concentrations in the coming years. Indeed, MPs have been detected across various environmental compartments, including soil [2], sediment [3], clouds [4], , wildlife [5], , human lungs [6], and most notably, in aquatic environments. An estimated 109 million metric tons (Mt) of plastics have accumulated in rivers and 30 Mt in oceans, comprising 88% macroplastics and 12% microplastics [7].*

L79-81: Change to “Firstly, secondly”.

Done.

L120: How many (proportion) Potential MPs were detected by ATR-FTIR.

100%

L162: First describe the shape, then talk about the polymer, and what about the concentration?

The paragraph was modified accordingly, and the order of the figures was modified. *Fibers and fragments were the most common particle shapes found at all sites and the order of importance changed among sites and between mesh sizes (Figs 2 and 3). Overall, more fiber and sphere particles were captured using the finer mesh size. Polyester, polyethylene, and polypropylene were the polymer types that were the most abundant in both mesh sizes at most site and accounted for over 85% of the total amount of particles overall (Figs 2 and 4). Many particles of nylon were found in one replicate at Portneuf accounting for ca. 60% of all particles.*

L203: Please bring appropriate references regarding your statement.

This reference was added. *38. Kye H, Kim J, Ju S, Lee, Lim C, Toon Y. Microplastics in water systems: A review of their impacts on the environment and their potential hazards. Heliyon. 9(3):e14359.*

L218: Plastics or microplastics; in China or in Chinese river?

This was revised to “the river”.

L228: In a previous study

Done.

L235: Please rewrite the sentence.

Done.

Figure 2 lacks a scale bar

For picture quality, it was decided to bring all MPs to a picture size that the reader could properly see. The shape and color are the two important criteria here as they are all MPs.

Figure 5 can add color to make it look better

The authors believe that adding 11 different color to code the sites will be too colorful and not add much scientific content so it was decided to let it as is.
