# Supplementary material for: Surface Water Microplastics in the St. Lawrence River and Estuary in Canada
Source: PLoS One. 2025 Apr 28;20(4):e0315739. doi: 10.1371/journal.pone.0315739 (PMC12036840; doi:10.1371/journal.pone.0315739)
Supplement: Attachment — Submitted filename: Langlois et al. Response to RE-reviews.docx [file pone.0315739.s006.docx]

**Comments to the Author**

1. If the authors have adequately addressed your comments raised in a previous round of review and you feel that this manuscript is now acceptable for publication, you may indicate that here to bypass the “Comments to the Author” section, enter your conflict of interest statement in the “Confidential to Editor” section, and submit your "Accept" recommendation.

Reviewer #1: (No Response)

Reviewer #2: All comments have been addressed

Reviewer #3: (No Response)

2. Is the manuscript technically sound, and do the data support the conclusions?

The manuscript must describe a technically sound piece of scientific research with data that supports the conclusions. Experiments must have been conducted rigorously, with appropriate controls, replication, and sample sizes. The conclusions must be drawn appropriately based on the data presented.

Reviewer #1: Yes Thanks!

Reviewer #2: Yes Thanks!

Reviewer #3: (No Response)

3. Has the statistical analysis been performed appropriately and rigorously?

Reviewer #1: I Don't Know

Reviewer #2: Yes Thanks!

Reviewer #3: (No Response)

4. Have the authors made all data underlying the findings in their manuscript fully available?

The [PLOS Data policy](https://can01.safelinks.protection.outlook.com/?url=http%3A%2F%2Ftrack.editorialmanager.com%2FCL0%2Fhttp%3A%252F%252Fwww.plosone.org%252Fstatic%252Fpolicies.action%2523sharing%2F1%2F010f0193629cb868-96d3598c-b672-48f4-a7d3-659cf034acc5-000000%2FNJX6Nm5TVVm2v5EQFYOYxpBSbdGKA_3M4tcuJYxOlEE%3D186&data=05%7C02%7CValerie.Langlois%40inrs.ca%7Cce16407055f34dfeaf3108dd0d49c798%7C80c9267a177f49439958b599ca0e54c2%7C0%7C0%7C638681332852396871%7CUnknown%7CTWFpbGZsb3d8eyJFbXB0eU1hcGkiOnRydWUsIlYiOiIwLjAuMDAwMCIsIlAiOiJXaW4zMiIsIkFOIjoiTWFpbCIsIldUIjoyfQ%3D%3D%7C0%7C%7C%7C&sdata=ehBmC7Aazfg2g6DAGFyv1GJT1OhVE%2BsIVgT6Bll7Ik4%3D&reserved=0) requires authors to make all data underlying the findings described in their manuscript fully available without restriction, with rare exception (please refer to the Data Availability Statement in the manuscript PDF file). The data should be provided as part of the manuscript or its supporting information, or deposited to a public repository. For example, in addition to summary statistics, the data points behind means, medians and variance measures should be available. If there are restrictions on publicly sharing data—e.g. participant privacy or use of data from a third party—those must be specified.

Reviewer #1: Yes Thanks!

Reviewer #2: Yes Thanks!

Reviewer #3: (No Response)

5. Is the manuscript presented in an intelligible fashion and written in standard English?

PLOS ONE does not copyedit accepted manuscripts, so the language in submitted articles must be clear, correct, and unambiguous. Any typographical or grammatical errors should be corrected at revision, so please note any specific errors here.

Reviewer #1: Yes Thanks!

Reviewer #2: Yes Thanks!

Reviewer #3: (No Response)

6. Review Comments to the Author

Please use the space provided to explain your answers to the questions above. You may also include additional comments for the author, including concerns about dual publication, research ethics, or publication ethics. (Please upload your review as an attachment if it exceeds 20,000 characters)

Reviewer #1: 1. Reviewer: How were blanks collected? E.g. distilled water poured onto net, then resuspended in the same way as samples? How much distilled water was poured onto net for each blank?

Response: A 1-L distilled water was poured into a glass on the boat and put at 4 oC until filtration. This has been specified in the text.

Reviewer: You should always refer to the specific line numbers where the changes were made. This will make your reviewers’ lives a lot easier.

**RE-Response:** We had submitted the track changes MS thinking it would be easier to track than giving the line numbers. We will be doing both this time.

2. Reviewer: Authors say that several digestion methods were tested; please list these methods somewhere in the manuscript or SI. Also remember to refer to the raw data in your repository.

Response: We have tested the digestion with hydroperoxide, H2O2 30 % (v/v) for different times and temperatures: 6 h at 65 oC, 24 h at 50 oC, 24 h at 25 oC, and 4 h at 50 oC with Fe(II).

This was adjusted in the text.

Reviewer: Line 132: “hydroperoxide” should be “hydrogen peroxide” (See [https://en.wikipedia.org/wiki/Hydroperoxide](https://can01.safelinks.protection.outlook.com/?url=https%3A%2F%2Fen.wikipedia.org%2Fwiki%2FHydroperoxide&data=05%7C02%7CValerie.Langlois%40inrs.ca%7Cce16407055f34dfeaf3108dd0d49c798%7C80c9267a177f49439958b599ca0e54c2%7C0%7C0%7C638681332852409384%7CUnknown%7CTWFpbGZsb3d8eyJFbXB0eU1hcGkiOnRydWUsIlYiOiIwLjAuMDAwMCIsIlAiOiJXaW4zMiIsIkFOIjoiTWFpbCIsIldUIjoyfQ%3D%3D%7C0%7C%7C%7C&sdata=9nGkBJXZy0bQDWNSXHAMqP5g4QtpmDBEq9bnokqyROc%3D&reserved=0)).

**RE-Response:** Changed accordingly. v

3. Reviewer: Fiber concentrations inferred using Manta net sampling is not accurate, because Manta nets underestimate particles smaller than its net mesh. For fibers, its diameter is typically 10-20 um, meaning it can slip through the manta net. Please add a line in your manuscript about how your fiber concentrations in this river system should be interpreted with caution because Manta nets are not designed to measure fibers.

Hung et al. (2020): “Capture of these particles is likely by random chance and is expected to be an underestimate of the total number of these small‐sized particles. For this reason, the quantities of particles with dimension smaller than the mesh size should be treated with caution and considered qualitative estimates.” [https://doi.org/10.1002/ieam.4325](https://can01.safelinks.protection.outlook.com/?url=https%3A%2F%2Fdoi.org%2F10.1002%2Fieam.4325&data=05%7C02%7CValerie.Langlois%40inrs.ca%7Cce16407055f34dfeaf3108dd0d49c798%7C80c9267a177f49439958b599ca0e54c2%7C0%7C0%7C638681332852421617%7CUnknown%7CTWFpbGZsb3d8eyJFbXB0eU1hcGkiOnRydWUsIlYiOiIwLjAuMDAwMCIsIlAiOiJXaW4zMiIsIkFOIjoiTWFpbCIsIldUIjoyfQ%3D%3D%7C0%7C%7C%7C&sdata=vo8HrYKeXneWqq0Vpe7Ms0a1w3%2FZtjvO9NH4YeXslZk%3D&reserved=0)

Also see Zhu et al. (2021): “Two surface water collection methods were employed: manta trawl (22,28) with a 333 μm mesh and 1 L grab. Manta samples (N = 65) were collected to quantify and characterize all microdebris, except for microfibers, which can slip through the holes in the mesh due to their shape.” [https://pubs.acs.org/doi/full/10.1021/acsestwater.0c00292](https://can01.safelinks.protection.outlook.com/?url=https%3A%2F%2Fpubs.acs.org%2Fdoi%2Ffull%2F10.1021%2Facsestwater.0c00292&data=05%7C02%7CValerie.Langlois%40inrs.ca%7Cce16407055f34dfeaf3108dd0d49c798%7C80c9267a177f49439958b599ca0e54c2%7C0%7C0%7C638681332852433675%7CUnknown%7CTWFpbGZsb3d8eyJFbXB0eU1hcGkiOnRydWUsIlYiOiIwLjAuMDAwMCIsIlAiOiJXaW4zMiIsIkFOIjoiTWFpbCIsIldUIjoyfQ%3D%3D%7C0%7C%7C%7C&sdata=4cp4y88SBontIq758kfGjB7jAXo69Kexu3sks0oHBsM%3D&reserved=0)

Response: Thanks for pointing this out. We have added your suggested comments to the text and use the Zhu et al. citation. Caution should also be considered when interpreting fiber MPs caught by the Manta as its underestimate particles smaller than its net mesh [45]. Therefore, the used of smaller mesh size nets like PolyMer (this study) is favored to improve fiber MP estimations.

Reviewer: Microfiber diameters tend to range between 10-20 um. They would still slip through your PolyMer net. I would remove your second sentence because it is not justified.

**RE-Response:** Thanks. We have changed it accordingly. Line number 283.

4. Reviewer: For results section, please add this basic information: average +/- SD for all 11 sites combined, which site had highest MP concentration (average +/- SD), which site had lowest MP concentration (average +/- SD). I’m not sure why you are using this new measure of concentration, “particles of plastic per million liters” or PPML; instead of inventing a new unit, please consider using existing units to make your study more comparable to others. See Bucci et al. (2020): usually, units of concentration used in ecotoxicology tests are “per mL” or “per L”. More and more often, ecotoxicologists are trying to use environmentally relevant concentrations in their experiments. To make their lives easier, and to harmonize reporting across studies, please consider reporting in a unit that other people use. Unless you provide justification for why you are using a new unit. Please report the average +/- SD concentration for your blanks. Did you blank correct?

Response: The information on average +/- SD for all 11 sites combined, which site had highest MP concentration (average +/- SD), which site had lowest MP concentration (average +/- SD) was added in the text: MP concentrations (mean +/- SD) for all 11 sites combined was 72.3 ± 93.0 PPML and was highest at the La Pocatière site (111.0 ± 120.0 PPML) and lowest at the Sorel site (38.7 ± 40.8 PPML).
This unit (PPML) was simply used because of the small number of MPs in the samples. Otherwise, the numbers would have been too long to report (too many 0.0000). The average +/- SD were added. Yes, we blank corrected.

Reviewer: The PPML still kind of bothers me, but I understand where the authors are coming from.

**RE-Response:** Good, thanks.

5. Reviewer: Lines 233-234: you say concentrations never exceed 0.001 particles per L – for surface water? And where – in US and Canada? Please make your writing a bit clearer, more informative. But in Rowenczyk et al. (2022), they found a maximum of 0.032 MPs per L in SLRE middle portion – contradiction? Also in Rowenczyk et al., they use particles/L. In order to make your results easily comparable to theirs, doesn’t it make sense to use the same units? I have not seen PPML used before in the literature. Two possibilities: 1) PPML has not been used before, then consider not creating a new unit; we already have a harmonization problem in the plastics field. 2) PPML has been used before: even if this is the case, it deviates from the norm and also makes harmonization difficult.

Response: Thanks for pointing this out, we have revamped the paragraph as: We expected to find higher MP concentrations in surface waters upstream than downstream considering the high population density living nearby the Great Lakes and SLRE from the United States and Canada, but lower concentrations were found than elsewhere. For example, Luo et al. (2019) measured concentrations around 2,000,000 PPML near major urban centers in China [46]. This difference can be explained by the hydrology and typology of the SLRE. Indeed, studies have shown that shoreline accumulation and deposition in the sediment were not considered as they can be collected and decontaminated [22]. Castañeda et al. (2014) have estimated MP loadings in SLRE sediment up to 3,800,000 PPML [22] at sites which have accumulated continuously for over 50 years compared to transitioning surface water [47]. In another study, fragments and fibers are the most prevalent particles found in the sediment [48] in South Africa, which could correlate with the variation in shape observed from the sampling campaign.The second part of the question was addressed above.

Reviewer: If you start your sentence with “We expected to find higher MP concentrations in surface waters upstream than downstream considering
the high population density living nearby the Great Lakes and SLRE from the United
States and Canada…” then I would expect you to end the sentence discussing what you actually found regarding the differences between upstream and downstream MP concentrations. Instead, you talk about an unconnected topic, which is how your concentrations are lower compared to other locations. Consider splitting into two separate sentences.

Comparing water to sediment doesn’t make sense – it’s like comparing apples to oranges.

This entire paragraph consists of a series of sentences with unrelated themes strung together. First you talk about surface water. Then you talk about sediment. Then you added a sentence about MP morphology, which has no connection to the previous sentences. I suggest separating the sentences to form their own paragraphs, so that each paragraph begins with a topic sentence and the rest of the paragraph expands on that topic sentence.

**RE-Response:** Thanks. We have removed the topic related on surface water to allow to compare other matrices in the flow. Line number 284.

***

I do not need to see the manuscript again. If all of the above changes are made, then I recommend this manuscript for publication.

**RE-Response:** Thanks for agreeing with all the changes made by the authors on the last round of reviews.

Reviewer #2: (No Response)

**RE-Response:** Thanks for agreeing with the lengthy responses made by the authors on the last round of reviews.

Reviewer #3: General Comment:
In the manuscript “PONE-D-24-18181R1,” the authors present a study on the presence and behavior of microplastics (MPs) on the water surface at 11 sites along the St. Lawrence River and Estuary in Canada. Using nets with different mesh sizes (100 µm and 300 µm) for collection and FTIR analysis, the authors identified MPs predominantly composed of fibers, fragments, and spheres, with polyester, polyethylene, and polypropylene being the most common polymers. The study also explores the relationship between salinity gradients and the aggregation of MPs, emphasizing the importance of this factor in estuarine systems. While the topic of the manuscript is relevant given the growing interest in MPs in estuarine environments and their potential environmental impacts, the study has significant deficiencies that justify a recommendation for rejection in a high-impact journal like PLOS ONE.

Specific Comments:

1. The scientific contribution is primarily incremental and lacks substantial innovation. The relationship between salinity and MP aggregation, which is a central focus of the study, has already been addressed in existing literature. The manuscript does not present a theoretical or experimental approach that meaningfully challenges or expands current understanding.

2. The study’s regional focus on the St. Lawrence River and Estuary limits its global applicability and reduces its appeal to a diverse international audience, which is the primary readership of PLOS ONE.

3. The introduction of the unit "particles per million liters" (PPML) is not convincingly justified and offers no clear advantage over widely accepted standardized units, such as particles per liter. This choice hampers comparability with global studies and compromises the harmonization of results within the field.

4. While the use of nets with different mesh sizes is methodologically interesting, it does not represent a significant advancement in the field. Instead, it highlights known limitations of sampling methods without proposing creative solutions or methodological innovations that advance the discipline. For a journal like PLOS ONE, studies are expected to contribute significantly to the progression of scientific knowledge through novel theories, innovative methodologies, or results with broad applicability.

5. Although the authors mention anti-contamination measures, their description is superficial and insufficient to inspire full confidence in the results. Essential details, such as whether laminar flow chambers were used during sample handling, the type of clothing worn by operators to prevent contamination with synthetic fibers (e.g., cotton or disposable lab coats), and the frequency of contamination controls in the laboratory, are not adequately discussed. Moreover, while field blanks are noted as a good practice, there is no detailed explanation of how these control values were used to adjust the reported concentrations of MPs in environmental samples.

6. The lack of comprehensive details about anti-contamination procedures undermines the methodological robustness of the study. Given the ubiquity of plastics in laboratory and field environments, studies on MPs require stringent safeguards against external contamination. Although the authors acknowledge the importance of preventing contamination, the superficiality of their descriptions compromises the transparency and reliability of their data, thereby calling into question the validity of their conclusions.

7. Thus, although the manuscript demonstrates technical merit, it fails to meet the criteria for theoretical impact and innovation expected for publication in a high-impact journal. I recommend that the authors consider submitting their study to a regional or specialized journal, where its incremental contributions may be better appreciated and appropriately valued.

**Response**: It is unfortunate to get a third reviewer seven months after first submission to PLOS ONE who is so negative. It has been months that the authors are redoing statistical analyses, graphs, maps, etc. to please the first two reviewers. The first two reviewers and the authors disagree with this new reviewer. The topic is timely and relevant to the readers of PLOS ONE. Plus, most of the new reviewer comments were already discussed by the previous reviewers.

7. PLOS authors have the option to publish the peer review history of their article ([what does this mean?](https://can01.safelinks.protection.outlook.com/?url=https%3A%2F%2Ftrack.editorialmanager.com%2FCL0%2Fhttps%3A%252F%252Fjournals.plos.org%252Fplosone%252Fs%252Feditorial-and-peer-review-process%2523loc-peer-review-history%2F1%2F010f0193629cb868-96d3598c-b672-48f4-a7d3-659cf034acc5-000000%2FqyFd9eXtIrtL4DdciJ4eIjhPT2Aj-FWZemap9PP1kP4%3D186&data=05%7C02%7CValerie.Langlois%40inrs.ca%7Cce16407055f34dfeaf3108dd0d49c798%7C80c9267a177f49439958b599ca0e54c2%7C0%7C0%7C638681332852446595%7CUnknown%7CTWFpbGZsb3d8eyJFbXB0eU1hcGkiOnRydWUsIlYiOiIwLjAuMDAwMCIsIlAiOiJXaW4zMiIsIkFOIjoiTWFpbCIsIldUIjoyfQ%3D%3D%7C0%7C%7C%7C&sdata=9gGrBNFMmBVpZn5YpoF721NMIYDgz0nTdHuk3HC2bjg%3D&reserved=0)). If published, this will include your full peer review and any attached files.

If you choose “no”, your identity will remain anonymous but your review may still be made public.

**Do you want your identity to be public for this peer review?** For information about this choice, including consent withdrawal, please see our [Privacy Policy](https://can01.safelinks.protection.outlook.com/?url=https%3A%2F%2Ftrack.editorialmanager.com%2FCL0%2Fhttps%3A%252F%252Fwww.plos.org%252Fprivacy-policy%2F1%2F010f0193629cb868-96d3598c-b672-48f4-a7d3-659cf034acc5-000000%2FTyPFvyL3RYyKAXsg006BGz_pikPW_nzI2R7ILmiVkNY%3D186&data=05%7C02%7CValerie.Langlois%40inrs.ca%7Cce16407055f34dfeaf3108dd0d49c798%7C80c9267a177f49439958b599ca0e54c2%7C0%7C0%7C638681332852459208%7CUnknown%7CTWFpbGZsb3d8eyJFbXB0eU1hcGkiOnRydWUsIlYiOiIwLjAuMDAwMCIsIlAiOiJXaW4zMiIsIkFOIjoiTWFpbCIsIldUIjoyfQ%3D%3D%7C0%7C%7C%7C&sdata=8DQdfgD2c31lXJZq%2FaNc45dDMmnAuYPqF2Wml6vbdl0%3D&reserved=0).

Reviewer #1: No

Reviewer #2: No

Reviewer #3: No

It is unfortunate the reviewers do not identify themselves.
